# IL-17A Can Promote *Propionibacterium acnes*-Induced Sarcoidosis-Like Granulomatosis in Mice

**DOI:** 10.3389/fimmu.2019.01923

**Published:** 2019-08-14

**Authors:** Jiacui Song, Mengmeng Zhao, Qiuhong Li, Liqin Lu, Ying Zhou, Yuan Zhang, Tao Chen, Danli Tang, Nianyu Zhou, Chengsheng Yin, Dong Weng, Huiping Li

**Affiliations:** ^1^School of Medicine, Soochow University, Suzhou, China; ^2^Department of Respiratory Medicine, School of Medicine, Shanghai Pulmonary Hospital, Tongji University, Shanghai, China

**Keywords:** *Propionibacterium acnes*, sarcoidosis, granuloma, IL-17A, mice

## Abstract

The etiology of sarcoidosis is unknown. In this study, *Propionibacterium acnes* (PA) was used to induce sarcoidosis-like granulomatous inflammation in a mouse model. Wild-Type (WT) C57BL/6 mice were divided into three groups: (1) WT-PA group; (2) WT-PA + Incomplete Freund's Adjuvant (IFA) group; and (3) WT-PBS group. Loose granuloma formation was observed in the lungs on day 56 in the WT-PA and WT-PA + IFA groups. The proportions of peripheral Th17 cells in the WT-PA (*p* = 0.0004) and WT-PA + IFA groups (*p* = 0.0005) were significantly higher than that in the WT-PBS group. The proportions of peripheral Treg cells in the WT-PA (*p* < 0.0001) and WT-PA + IFA groups (*p* < 0.0001) were lower than that in the WT-PBS group. Then, to explore the mechanism of IL-17, Wild-Type (WT) C57BL/6 mice were divided into three groups: (1) WT-PBS group (2) WT-PA group; (3) WT-PA + mouse IL-17A neutralizing antibody (IL-17Ab) group. IL-17A gene knockout mice (KO) were divided into two groups: (1) KO -PA group; (2) KO-PBS group. The KO-PA and WT-PA + IL-17Ab groups showed reduced inflammation and no loose granuloma formation on day 56. As compared to the WT-PA group, the ratio of peripheral Th17 in the KO-PA (*p* < 0.0001) and WT-PA + IL-17Ab groups (*p* < 0.0001) decreased, while the ratio of peripheral Treg in the KO-PA (*p* < 0.0001) and WT-PA + IL-17Ab (*p* = 0.0069) groups increased on day 56. Hence, PA can be used to establish a mouse model of sarcoidosis-like granuloma. IL-17A plays an important role in experimental sarcoidosis-like granuloma formation.

## Introduction

Sarcoidosis, a systemic granulomatous disease of unknown etiology, is characterized by non-caseous necrotic granulomata (1). The pathogenesis of sarcoidosis remains unclear, and may be associated with infection (2), genetics (3), immunity (4), and environmental exposure (5). Previous studies have indicated that *Propionibacterium acnes* (PA) might be a causative pathogen for sarcoidosis (6–8).

Since the cause of sarcoidosis is unknown, a standardized animal model is lacking. Since 1998, PA has been used to successfully establish an animal model of liver granuloma (9). Ichiyasu et al. (10) used PA to induce sarcoidosis-like granulomatosis in rabbit lung. However, whether existing animal models of sarcoidosis-like granulomatosis can be used to investigate sarcoidosis in humans remains unknown. In addition, there is no consensus on the dosage, time and route of stimulant administration to establish animal models of sarcoidosis-like granulomatosis.

In the current study, we used inactivated PA with or without incomplete Freund's adjuvant (IFA) to perform intraperitoneal pre-sensitization, followed by multiple low-dose intratracheal inoculations of inactivated PA to establish a mouse model of sarcoidosis-like granulomatosis. In addition, we also performed a long-term observation of granuloma dissipation in the mouse model. We aimed to establish a simple and practical mouse model of sarcoidosis-like granulomatosis that resembles human sarcoidosis, and used IL-17A^−/−^ mice and IL-17A neutralizing antibody to further investigate the role of IL-17A in sarcoidosis granuloma development.

## Materials and Methods

### Experimental Animals

Specific pathogen-free (SPF) female C57BL/6 mice (Shanghai SLAC Laboratory Animal Co., Ltd.) and female IL-17A knockout mice (IL-17A^−/−^) (Tokyo University of Science) were maintained up to 6–8 weeks of age, with free access to water and food. All animal handling and experimental procedures were approved by the Experimental Animal Center of Tongji University (No. K17-016).

### Reagents and Instruments

PA (purchased from ATCC, USA, batch number 6919) was cultured in Clostridium Perfringens medium at 37°C for 48 h. The bacterial suspension of PA was prepared, and then PA was inactivated at 65°C for 30 min. The OD600 of the PA suspension was measured using a spectrophotometer (BioTek Epoch2). Mouse IL-17A neutralizing antibody was purchased from BioXcell (BP0173-5MG).

### Mouse Model Establishment

Wild-type C57BL/6 mice were randomized into three groups (Figure S1A): WT-PA group (*n* = 114), in which mice were pre-sensitized by intraperitoneal injection of inactivated PA (0.25 mL, 2 mg/mL) and then intratracheally inoculated with inactivated PA (0.05 mL, 10 mg/mL) at day 14, 28, and 42 after the pre-sensitization; WT-PA + IFA group (*n* = 42), in which mice were pre-sensitized by intraperitoneal injection of inactivated PA plus IFA (0.25 mL, 2 mg/mL) and then received intratracheal inoculation in the same manner as the WT-PA group; and WT-PBS group (*n* = 42), in which mice received intraperitoneal injection of PBS (0.25 mL) and intratracheal inoculation of PBS (0.05 mL) in the same manner as the WT-PA group. Peripheral blood, BALF, and lung tissue samples of each group were collected on day 15, 17, 19, 21, 28, 42, and 56, respectively.

Our preliminary results showed that the two modeling methods (with or without IFA) had similar effects on mouse model establishment. Hence, we did not use IFA in the final experiments. The WT-PA group was further divided into two groups (Figure S1B): WT-PA-A group (*n* = 36), in which mice were intratracheally inoculated with PA at day 56, 70, 84, 98, 112, and 126 after the pre-sensitization in addition to the earlier PA inoculations, and the WT-PA-B group (*n* = 36), in which mice did not received extra intratracheal inoculation. The lung tissue samples were collected on day 70, 84, 98, 112, 126, and 140, respectively, to observe granuloma dissipation in the lung tissue.

IL-17A^−/−^ mice were used to investigate the role of IL-17A in PA-induced granulomatosis (Figure S1C). The IL-17A^−/−^ mice were divided into the KO-PA group (*n* = 24), in which the mice were intratracheally inoculated in the same manner as the WT mice, and the KO-PBS group (*n* = 24), in which the mice were intratracheally inoculated with PBS. In the WT-PA + IL-17Ab group (*n* = 24), the WT mice were pre-sensitized with intraperitoneal injection of inactivated PA (0.25 mL, 2 mg/mL), and then intraperitoneally injected with IL-17Ab (30 μg/20 g) at day 14 after the pre-sensitization, and intratracheally inoculated with inactivated PA (0.05 mL, 10 mg/mL) 4 h after the IL-17Ab injection. Then the mice were challenged with inactivated PA on day 28 and 42 after the pre-sensitization. Peripheral blood, BALF, and lung tissue samples were collected on day 17, 21, 28, and 56, respectively.

### Hematoxylin and Eosin Staining

Lung tissue samples were fixed with universal tissue fixative (4% paraformaldehyde, neutralized) for 24 h and then paraffin-embedded for sectioning. The tissue sections were stained with hematoxylin and eosin. The stained tissue sections were scanned with Leica pathological slice scanner (LEICA SCN400). Two experienced pathologists independently reviewed the staining results.

### Immunohistochemistry

Lung tissue sections were incubated with the primary antibodies anti-CD4 (Servicebio, GB11064) at 1:1,000 and anti-CD68 (Servicebio, GB11067) at 1:300, and then incubated with the HRP-goat anti-rabbit secondary antibody (Servicebio, GB21303) at 1:200 dilution. Color was developed by DAB. The tissue sections were observed under a microscope.

### Bacterial Staining

Lung tissue samples were fixed with universal tissue fixative (neutral) for 24 h, embedded in paraffin, and sectioned. Gram staining was performed on the tissue sections. The stained tissue section was observed under a microscope.

### Bronchoalveolar Lavage Collection

Bronchoalveolar lavage (BAL) cells were collected by five injections of 0.8 ml of sterile PBS containing 2% fetal calf serum (Sigma, St. Louis, MO) and 2 mmol/L ethylenediaminetetraacetic acid. The total number of BAL cells was counted with a hemocytometer and used for flow cytometry detection. The supernatants from BAL were used for ELISA detection.

### Flow Cytometry

The cells were resuspended in FACS solution and the cell suspension was divided into three aliquots for the following antibodies: (1) surface-staining with anti-mouse CD4 antibody followed by permeabilization and staining with anti-mouse IFN-γ and IL-17A antibodies; (2) surface staining with anti-mouse CD4 and CD25 antibodies followed by permeabilization and staining with anti-mouse Foxp3 antibody; (3) surface staining with anti-mouse CD3, CD4, and CD8 antibodies. All fluorescent antibodies were purchased from eBioscience (San Diego, CA). The stained cells were analyzed by flow cytometry (Beckman Coulter). Mouse peripheral blood was collected, and peripheral mononuclear cells were separated after lysing the red blood cells.

### Cytokine Analysis

BALF supernatant was collected from at least five mice in each group and analyzed by ELISA (Neobioscience) to determine IL-17A, IL-23, TGF-β1, and IL-10 levels.

### Statistical Analysis

The Graphpad Prism 6 software was used for statistical analysis. Continuous variables were presented as mean ± standard deviation. Multi-group comparison was analyzed by one-way ANOVA followed by Tukey test. *P* < 0.05 was considered statistically significant.

## Results

### Establishment of a Mouse Model of Sarcoidosis-Like Granulomatosis

**Figure 1:**
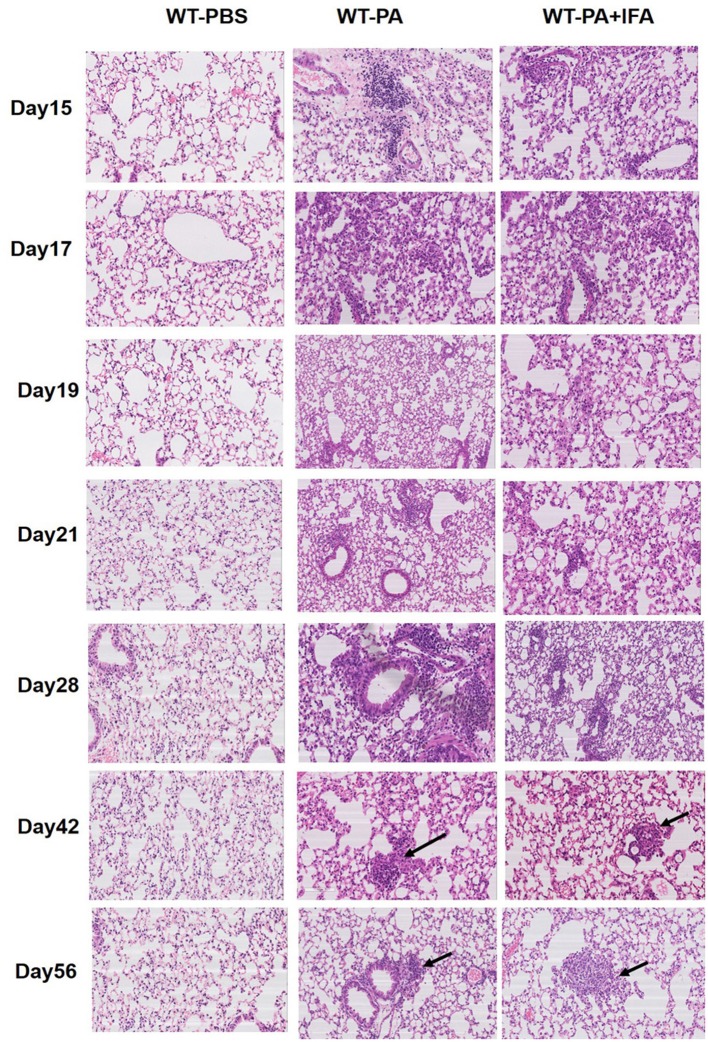
Representative images of hematoxylin and eosin staining of mouse lung tissue specimens. Lung tissue specimens were collected on the 15th, 17th, 19th, 21st, 28th, 42nd, 56th day (*n* = 6 each group). H and E staining was performed. The WT-PA and WT-PA + IFA groups show lymphocyte accumulation in the bronchial areas and chronic inflammation, small amount of macrophages (arrows) accumulated in the bronchial areas and immature granuloma on the 42nd day, and loose granuloma (arrows) on the 56th day. The images are 200×.

Figure 1 presents the results of hematoxylin and eosin staining of lung tissue specimens. Both the WT-PA and WT-PA + IFA groups showed lymphocyte accumulation in the bronchial areas and chronic inflammation on day 15, 17, 19, 21, and 28. Macrophage accumulation in the bronchial areas and immature granuloma were seen on day 42, and loose granuloma in the bronchial and blood vessel areas was seen on day 56 (higher magnification of the areas with the arrows was shown in Figure S5A). The WT-PBS group showed no such features. Loose granulomatous tissue could be seen in the lung of mice from the WT-PA and WT-PA + IFA groups. This means that the two modeling methods (with or without IFA) have similar effects on pathology in mouse model establishment.

**Figure 2:**
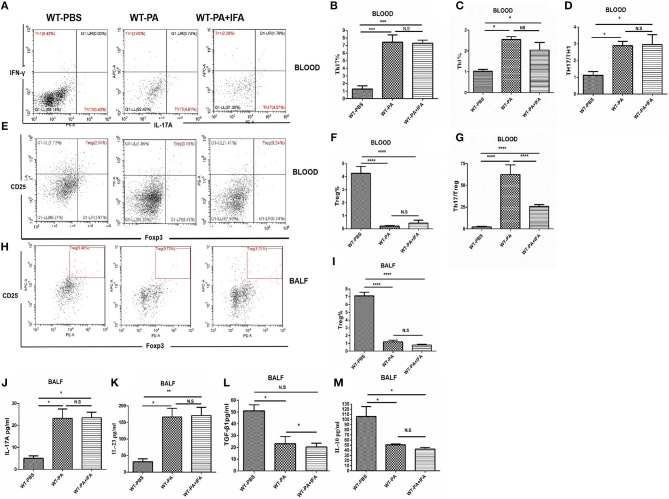
Flow cytometry analysis and ELISA in peripheral blood and BALF. On the 56th day, peripheral blood and BALF samples were collected from the 3 groups and mononuclear cells were harvested. **(A)** Blood CD4+ lymphocytes were enriched with FITC-anti-CD4. Anti-INFγ and anti-IL-17A were used to label TH1 and TH17 cells. **(B)** Peripheral Th17 cell%. **(C)** Th1 cell%. **(D)** Th17/Th1 ratio. **(E)** Blood CH4+ cells were selected. Treg cells were labeled with anti-CD25 and anti-Foxp3 **(F)** Peripheral Treg cells. **(G)** Th17/Treg ratio. **(H)** BALF CH4+ cells were selected. Treg cells were labeled with anti-CD25 and anti-Foxp3. **(I)** BALF Treg cells. ELISA was performed to analyze cytokine levels in BALF including **(J)** IL-17A, **(K)** IL-23, **(L)** TGF-β1, **(M)** IL-10. ^*^P < 0.05, ^**^P < 0.01, ^***^P < 0.001, ^****^P < 0.0001. NS, not significant.

The proportions of Th1, Th17 (Figure 2A) and Treg cells (Figures 2E,H), and CD4/CD8 ratio (Figures S2A,C) in the peripheral blood and BALF samples collected on day 56 were analyzed by flow cytometry. The peripheral blood samples of the WT-PA and WT-PA + IFA groups had significantly increased Th17 cells% (Figure 2B, WT-PA vs. WT-PBS: *p* = 0.0004; WT-PA + IFA vs. WT-PBS: *p* = 0.0005), Th1 cells% (Figure 2C, WT-PA vs. WT-PBS: *p* = 0.0110; WT-PA + IFA vs. WT-PBS: *p* = 0.0356) and Th17/Th1 ratio (Figure 2D, WT-PA vs. WT-PBS: *p* = 0.0243; WT-PA + IFA vs. WT-PBS: *p* = 0.0211), significantly reduced Treg cells% (Figure 2F, WT-PA vs. WT-PBS: *p* < 0.0001; WT-PA + IFA vs. WT-PBS: *p* < 0.0001), and significantly elevated Th17/Treg ratio (Figure 2G, WT-PA vs. WT-PBS: *p* < 0.0001; WT-PA + IFA vs. WT-PBS: *p* < 0.0001) as compared to the WT-PBS group. The BALF samples of both PA groups showed significantly reduced Treg cells% (Figure 2I, WT-PA vs. WT-PBS: *p* < 0.0001; WT-PA + IFA vs. WT-PBS: *p* < 0.0001) as compared to the WT-PBS group. Th17%, Th1%, Th17/Th1 ratio, and Treg% of peripheral blood had no statistical difference (Figures 2B–D,F), but Th17/Treg ratios were statistically different between the WT-PA group and the WT-PA + IFA group (Figure 2G). The CD4/CD8 ratio in both PA groups were significantly reduced (Figure S2B) in the peripheral blood but significantly increased (Figure S2D) in the BALF as compared to the WT-PBS group. The total cell numbers and Lymphocytes numbers in both PA groups were increased in BALF samples on the 56th day (Figures S4A,B) as compared to the WT-PBS group.

The BALF collected on day 56 from both the WT-PA and WT-PA + IFA groups showed significantly higher levels of Th17-associated inflammatory factors, including IL-17A (Figure 2J, WT-PA vs. WT-PBS: *p* = 0.0203; WT-PA + IFA vs. WT-PBS: *p* = 0.0324) and IL-23 (Figure 2K, WT-PA vs. WT-PBS: *p* = 0.0095; WT-PA + IFA vs. WT-PBS: *p* = 0.0115) as compared to the WT-PBS group. In contrast, the levels of Treg-associated inflammatory factors, including TGF-β1 (Figure 2L, WT-PA vs. WT-PBS: *p* = 0.0163; WT-PA + IFA vs. WT-PBS: *p* = 0.0107) and IL-10 (Figure 2M, WT-PA vs. WT-PBS: *p* = 0.0331; WT-PA + IFA vs. WT-PBS: *p* = 0.0179) were significantly lower in the two PA groups as compared to the WT-PBS group. There was no significant difference in IL-17A, IL-23, and IL-10 in BALF collected on day 56 (Figures 2J,K,M), but there was significant difference in TGF-β1 (Figure 2L) in the two PA groups.

### Characterization of the Mouse Model of PA-Induced Granulomatosis

**Figure 3:**
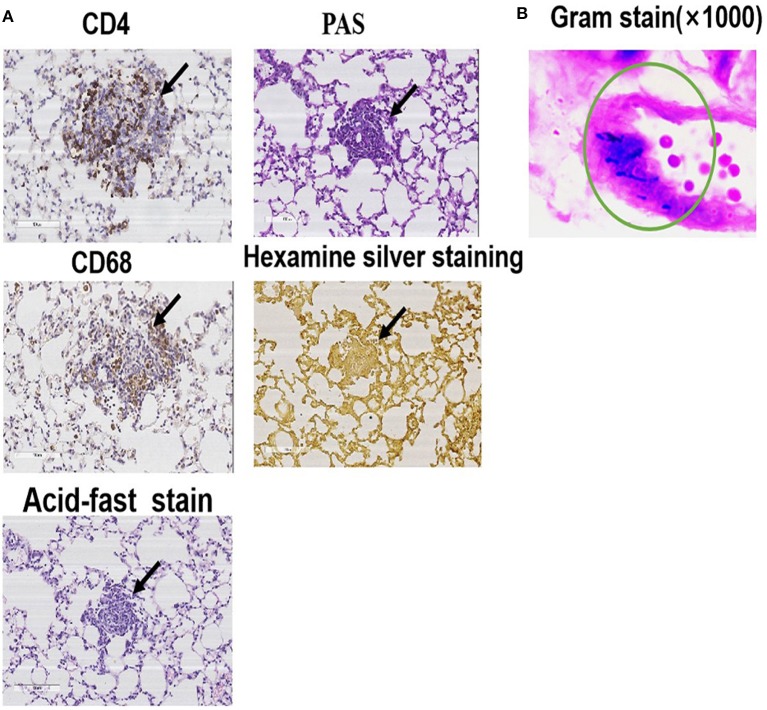
Representative images of immunohistochemical, PAS, acid-fast, and hexamine silver staining. **(A)** Lung tissue specimens were collected on the 56th day and used for immunohistochemical staining with anti-CD4 and anti-CD68, PAS, acid-fast, and hexamine silver. **(B)** Gram staining to detect PA.

Immunohistochemical, hexamine silver, and acid-fast staining of the lung tissue specimens on day 56 showed positive CD4 and CD68 but negative PAS, hexamine silver, and acid-fast (Figure 3A). These results indicated that the mouse model of PA-induced sarcoidosis-like granulomatosis has similar lung tissue characteristics as human granulomatosis, and fungal and tuberculosis bacterial infection can be excluded. The lung tissue sections also showed weak Gram-positive staining (Figure 3B), suggesting the presence of PA in the lung granulomatous tissues.

The WT-PA-A group, which received extra PA stimulation at day 70, 84, 98, 112, and 126, demonstrated sustained granulomatosis and even developed fibrotic lesions; whereas the control WT-PA-B group, which received no further PA stimulation after day 56, showed gradually dissipated granuloma and interstitial fibrosis (Figure S3A). Th17 cells% (Figure S3B) and Th17/Treg ratio (Figure S3C) in peripheral blood remained at high levels in the WT-PA-A group but reduced gradually in the WT-PA-B group.

### IL-17A Knockout and IL-17A Neutralizing Antibody Treatment Alleviated Lung Inflammation in Mice With PA-Induced Granulomatosis

**Figure 4:**
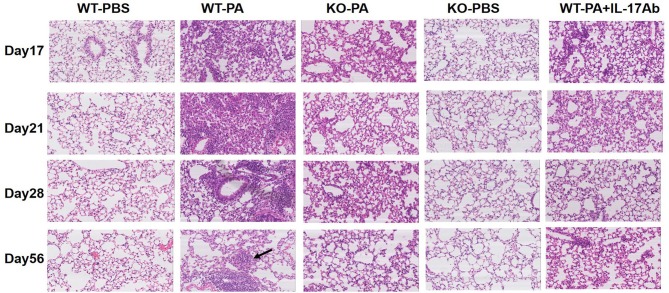
Representative images of HandE staining of the WT-PBS, WT-PA, KO-PA, KO-PBS, and WT-PA + IL-17Ab groups. Lung tissue specimens from the five groups (*n* = 6 each group) were collected on the 17th, 21st, 28th, and 56th day and stained. The KO-PA and WT-PA + IL-17Ab groups show less inflammatory cell infiltration on the 17th, 21st, 28th day than the WT-PA group and no loose granuloma on the 56th day.

The KO-PA and WT-PA + IL-17Ab groups showed reduced inflammatory cell infiltration in the lung tissues on day 17, 21, and 28, and no loose granuloma on day 56 as compared to the WT-PA group (Figure 4 and higher magnification in Figure S5B).

**Figure 5:**
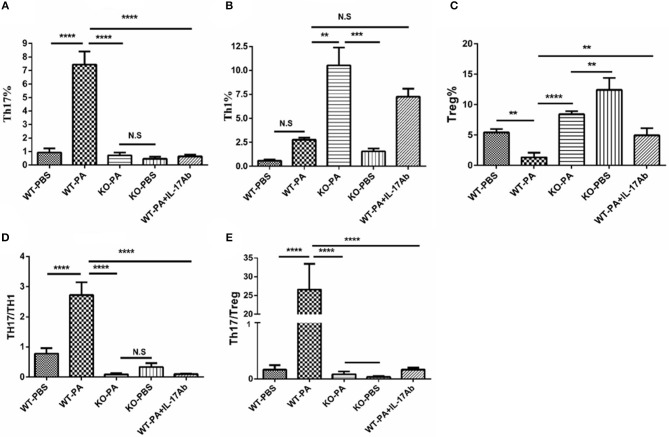
Flow cytometry analysis of mononuclear cells in peripheral blood samples. Peripheral blood samples were collected on the 56th day and mononuclear cells were harvested. CD4+ lymphocytes were enriched and anti-INFγ and anti-IL-17A antibodies were used to label TH1 and TH17 cells. **(A)** Th17%, **(B)** Th1%, **(C)** Treg%, **(D)** Th17/Th1 ratio, and **(E)** Th17/Treg ratio were analyzed. ^**^P < 0.01, ^***^P < 0.001, ^****^P < 0.0001. NS, not significant.

As compared to the WT-PA group, the KO-PA and WT-PA + IL-17Ab groups had significantly reduced Th17% in the peripheral blood collected on day 56 (Figure 5A, WT-PA vs. KO-PA: *p* < 0.0001; WT-PA vs. WT-PA + IL-17Ab: *p* < 0.0001), significantly higher Th1% (Figure 5B, WT-PA vs. KO-PA: *p* = 0.0025; WT-PA vs. WT-PA + IL-17Ab: *p* = N.S) and Treg cells% (Figure 5C, WT-PA vs. KO-PA: *p* < 0.0001; WT-PA vs. WT-PA + IL-17Ab: *p* = 0.0069), and significantly lower Th17/Th1 (Figure 5D, WT-PA vs. KO-PA: *p* < 0.0001; WT-PA vs. WT-PA + IL-17Ab: *p* < 0.0001) and Th17/Treg ratios (Figure 5E, WT-PA vs. KO-PA: *p* < 0.0001; WT-PA vs. WT-PA + IL-17Ab: *p* < 0.0001).

**Figure 6:**
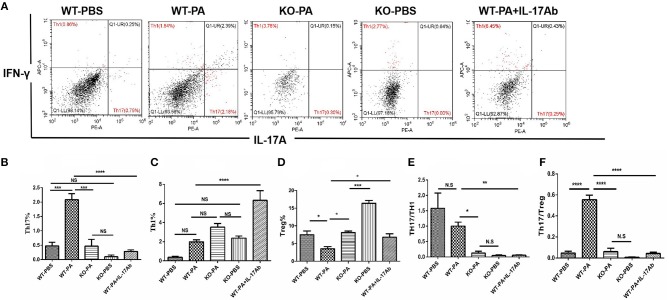
Flow cytometry analysis of mononuclear cells in the BALF samples. **(A)** BALF samples were collected on the 56th day and mononuclear cells were harvested. CD4+ lymphocytes were enriched and anti-INFγ and anti-IL-17A antibodies were used to label TH1 and TH17 cells. **(B)** Th17%, **(C)** Th1%, **(D)**Treg%, **(E)** Th17/Th1 ratio, and **(F)** Th17/Treg ratio were analyzed. ^*^P < 0.05, ^**^P < 0.01, ^***^P < 0.001, ^****^P < 0.0001. NS, not significant.

Results of flow cytometry analysis of the BALF samples collected on day 56 are presented in Figure 6A. As compared to the WT-PA group, the KO-PA and WT-PA + IL-17Ab groups had significantly lower Th17% (Figure 6B, WT-PA vs. KO-PA: *p* = 0.0003; WT-PA vs. WT-PA + IL-17Ab: *p* < 0.0001), higher Th1% (Figure 6C, WT-PA vs. KO-PA: *p* = N.S; WT-PA vs. WT-PA + IL-17Ab: *p* < 0.0001) and Treg% (Figure 6D, WT-PA vs. KO-PA: *p* = 0.0124; WT-PA vs. WT-PA + IL-17Ab: *p* = 0.0448), and lower Th17/Th1 (Figure 6E, WT-PA vs. KO-PA: *p* = 0.0186; WT-PA vs. WT-PA + IL-17Ab: *p* = 0.0015) and Th17/Treg (Figure 6F, WT-PA vs. KO-PA: *p* < 0.0001; WT-PA vs. WT-PA + IL-17Ab: *p* < 0.0001) ratios. The total cell numbers and Lymphocytes numbers in KO-PA and WT-PA + IL-17Ab groups were decreased in BALF samples on the 56th day (Figures S4C,D) as compared to the WT-PA group.

**Figure 7:**
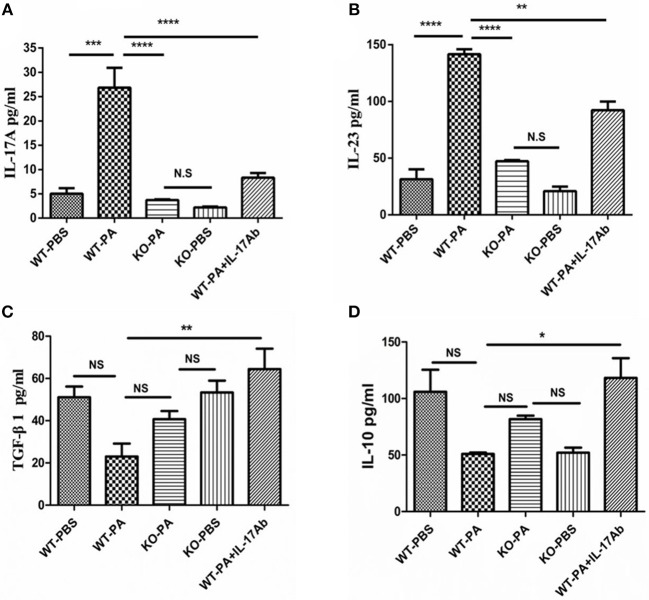
ELISA detection for inflammatory cytokines in BALF. BALF was collected on the 56th day. The levels of **(A)** IL-17A, **(B)** IL-23, **(C)** TGF-β1, and **(D)** IL-10 in BALF were measure by ELISA. ^*^P < 0.05, ^**^P < 0.01, ^***^P < 0.001, ^****^P < 0.0001. NS, not significant.

As compared to the WT-PA group, the KO-PA and WT-PA + IL-17Ab groups showed significantly lower levels of Th17-associated inflammatory factors in the BALF collected on day 56, including IL-17A (Figure 7A, WT-PA vs. KO-PA: *p* < 0.0001; WT-PA vs. WT-PA + IL-17Ab: *p* < 0.0001) and IL-23 (Figure 7B, WT-PA vs. KO-PA: *p* < 0.0001; WT-PA vs. WT-PA + IL-17Ab: *p* = 0.0015), but higher levels of Treg-associated inflammatory factors including TGF-β1 (Figure 7C, WT-PA vs. KO-PA: *p* = N.S.; WT-PA vs. WT-PA + IL-17Ab: *p* = 0.0067) and IL-10 (Figure 7D, WT-PA vs. KO-PA: *p* = N.S.; WT-PA vs. WT-PA + IL-17Ab: *p* = 0.0217).

## Discussion

The etiology and pathogenesis of sarcoidosis remain unclear. Animal models of sarcoidosis are critical to advance our understanding of the disease, and those that can represent the characteristics of human sarcoidosis are a research hotspot.

Some new animal models have been recently described that may be useful tools to fill the critical knowledge gaps (11). Chen et al. intraperitoneally injected *M. tuberculosis* whole cell lysate (Mtb-WCL) or recombinant *M. tuberculosis* catalase–peroxidase (mKatG) to induce granulomas in female Lewis rats or C57BL/6 mice. However, lack of direct clinical correlation in the induction of granulomas by conjugated beads may limit generalized use of this model (12). In addition to whole-cell lysates and mKatG, mycobacterial superoxide dismutaseA (sodA), which was previously linked to sarcoidosis granulomas, was used to generate animal models of sarcoidosis. The model's histological and immunological similarities to human sarcoidosis and lack of reliance on co-administered dendritic cells (DCs) make it potentially promising (13). Sensitization and challenge using heat-killed P. acnes and DCs were performed to induce pulmonary granulomatosis in C57BL/6 mice (14). Herndon et al.'s model of urease coupled to microbeads induced sarcoidosis-like granulomas in rats suggesting that urease alone is insufficient to trigger disease. They identified a potential role of urease in granuloma formation (15). However, a commonly accepted animal model of sarcoidosis is lacking.

Previously, sarcoidosis tissue extract (Kveim antigen) was used for animal experiments (16). However, due to the unclear composition of Kveim antigen, challenges in access to Kveim antigen, and wide variation in the types of experimental animals and inoculation route and dose, the Kveim antigen method often leads to inconsistent results and is not widely adopted. Complete Freund's adjuvant (CFA) was also used to establish an animal model of sarcoidosis. Bergeron et al. (17) injected CFA into the tail vein of Wistar rats to develop a rat model of granulomatosis. Since the mechanism of action of CFA-induced granulomatosis is unclear, the CFA injection method is not commonly adopted. *Mycobacterium tuberculosis* (MTB), which may cause sarcoidosis (18, 19), was used to develop animal models of MTB-induced sarcoidosis. Drake et al. found Mycobacterium 16S rRNA and rpoB sequences in sarcoidosis tissue specimens (20). However, previous studies have shown that MTB positive rate is substantially lower in sarcoidosis tissue specimens than in tuberculosis tissue specimens, which indicates that MTB in sarcoidosis tissue specimens might be background bacteria but not a causative pathogen for sarcoidosis (21). Liu performed Warthin-Starry silver staining to discover obvious PM2.5 and a small amount of silica dust particles in the granuloma cells of sarcoidosis specimens (22). Whether the findings from Liu's study can be used as a theory to develop animal models of sarcoidosis remains unknown.

Recent studies have demonstrated that PA is associated with sarcoidosis (2, 8, 23). Werner et al. generated a mouse model of early granuloma formation through intratracheal administration of live P. acnes directly into the lungs of mice. These mice develop pulmonary granulomas with no experimental manipulation of P. acnes itself or the immune system, such as adjuvants or multiple sensitizations (24). However, whether existing animal models of sarcoidosis-like granulomatosis can be used to investigate sarcoidosis in humans remain unknown. Jiang et al. investigated whether repeated challenge with PA induces persistent inflammation leading to sarcoidosis followed by Pulmonary fibrosis (PF) in mice. Inflammation, granulomata, and features of fibrosis were evaluated every 7 days until day 70. Repeated boosting with PA to induce PF might be a useful model for future studies of sarcoidosis-associated PF (25). Both Jiang's study and our study used heat-killed PA to stimulate female mice. However, stimulation times of heat-killed PA were different (two times in Jiang's study and three times in our study). The longest observation time was different (70 days in Jiang's study and 140 days in our study). For the control group (remission group), the method and observation time were also different.

In this study, we used multiple intratracheal inoculations of low-dose PA to establish a mouse model of sarcoidosis-like granulomatosis. We observed this model for up to 140 days. The advantage of this model is that it mimics the hispathological and immunological characteristics of the inflammation stage of human sarcoidosis. In contrast to the previously reported paw pad injection (26) and vein injection (27), we performed peritoneal pre-sensitization followed by intratracheal inoculation of inactivated PA. The intratracheal inoculation of inactivated PA resulted in obvious loose granuloma in lung tissues regardless of the addition of IFA (Figure 1). Thus, we used multiple intratracheal inoculations of low-dose inactivated PA without IFA to establish the mouse model in this study. The advantage of this mouse model is that it mimics the histopathological and immunological characteristics of the inflammation stage of human sarcoidosis-like granulomatosis, which resembles the development of human chest sarcoidosis. Thus, the mouse model in the current study may be a suitable animal model to investigate human sarcoidosis. In addition, previous studies on animal model development usually only observed the model for a short time and the granuloma disappeared at late stage (28). To observe the long-term effects of the PA inoculation, in the current study, we divided the WT-PA group into the WT-PA-A group, which received continuous inoculation and was observed for granuloma progression, and the WT-PA-B group, which received no extra inoculation and was observed for granuloma dissipation. The WT-PA-A group showed persistent granuloma and even fibrotic lesions, while the WT-PA-B group showed granuloma dissipation and gradual disappearance of interstitial fibrosis (Figure S3).

The pathogenic mechanisms underlying sarcoidosis are very complex, and multiple types of cells and cytokines are involved in sarcoidosis initiation. Th17/Treg cell imbalance may play an important role in the initiation and development of sarcoidosis (29, 30). Idali et al. found that Treg cell count was reduced in peripheral blood and BALF during active sarcoidosis, and proposed that the reduction in Treg cells and loss of Treg cell function could lead to sarcoidosis development (31). Previous studies have shown that Th17% significantly increased in the peripheral blood and BALF of patients with active sarcoidosis (32, 33). Patients with sarcoidosis have higher Th17 cells in the peripheral blood as compared to healthy controls (34). Th17 cells and γδ T cells are the main source for producing IL-17A. We went through relevant literatures, and found that there were few clinical studies on γδ T cells in sarcoidosis (35, 36). In recent years, researchers mainly focused on Th17 cells in sarcoidosis. However, we cannot rule out the role of γδ T cells in sarcoidosis mice model. We also believe that γδ T cells may play some role in sarcoidosis and we may investigate the role of γδ T cells in future study. IL-17 A/IL-17R signaling played an important role in pulmonary inflammation through recruitment of neutrophils (37). These suggest that neutrophils might have some potential role in sarcoidosis, which needs further studies. Elevation of IL-17A levels has been found in patients with sarcoidosis (38, 39). IL-17A is the predominant pro-inflammatory cytokine secreted by Th17 cells. Hence, we investigated the role of IL-17 in our mice model using IL-17A^−/−^ mice and IL-17A neutralizing antibody. In our preliminary experiments, we used isotype mouse IgG1 (WT-PA-isotype control group) and found no difference in lung histopathology and inflammatory factors as compared to the WT-PA group (propionibacterium acne model group) (data not shown). Therefore, we excluded the non-specific combination of IgG1 and anti-IL-17A antibody in this study. Hence, isotype control IgG1 group was not included in the final experiment.

Hawkins et al. found that sarcoidosis CD4+ T cells exhibited loss of cellular function during progressive disease that follows the archetype of T cell exhaustion (40). They also demonstrated that CD4+ anergic responses to polyclonal TCR stimulation were present peripherally and within the lungs of sarcoid patients (41). These findings suggested normalized CD4(+) T cell function as a potential therapeutic target for sarcoidosis resolution.

In this study, we detected CD4+IFN-γ+ Th1 cells by flow cytometry. However, we did not detect IFN-γ and TNF-α expression by ELISA in this experiment. In this study, we mainly focused on Th17 and Treg-related cytokines (IL-17A, IL-23, TGF-β1, IL-10). However, IFN-γ and TNF-α are important cytokines for granuloma formation. We will include detection of IFN-γ and TNF-α in our future study.

Jiang et al. (25) found that TGF-β1 was elevated in a similar model, which is not consistent with our data. We speculate that the difference in TGF-β1 may be caused by different modeling methods between our study and Jiang's study. Our challenge on day 42 might lead to sustained stimulation, which might disturb the balance between pro-inflammatory cytokines and anti-inflammatory cytokines. The level of TGF-β1 might be inhibited by sustained activation of pro-inflammatory cytokines such as IL-17A and IL-23. Meanwhile, the decreased TGF-β1 level in WT-PA group were consistent with the tendency of decreased Tregs. However, the latent mechanism needs further investigation.

In the current study, the mouse model showed elevated peripheral Th17% and IL-17A levels in BALF (Figure 2). IL-17A knockout and IL-17A neutralizing antibody alleviated inflammation in the lung of mice with PA-induced granulomatosis, blocked loose sarcoidosis-like granuloma development, and reduced inflammatory factor levels in peripheral blood and BALF. These results suggested that IL-17A may be an important pro-inflammatory factor in PA-induced sarcoidosis-like granulomatosis.

In summary, a mouse model of PA-induced sarcoidosis-like granulomatosis was successfully established in this study. IL-17A may play an important pro-inflammatory role in the development of sarcoidosis-like granulomatosis.

## Ethics Statement

The study was approved by the institutional ethics committee of Shanghai Pulmonary Hospital (No. K17-016).

## Author Contributions

HL, DW, JS, MZ, and QL: experimental design. JS, MZ, QL, YZho, YZha, TC, DT, LL, NZ, CY, DW, and HL: data acquisition and analysis. JS, MZ, and HL: writing the manuscript. All authors read and approved the final manuscript.

## Conflict of Interest Statement

The authors declare that the research was conducted in the absence of any commercial or financial relationships that could be construed as a potential conflict of interest.
