# Supplementary material for: IL-17A Can Promote Propionibacterium acnes-Induced Sarcoidosis-Like Granulomatosis in Mice
Source: Front Immunol. 2019 Aug 14;10:1923. doi: 10.3389/fimmu.2019.01923 (PMC6702313; doi:10.3389/fimmu.2019.01923)
Supplement: Supplementary file 1 [file Data_Sheet_1.doc]

**
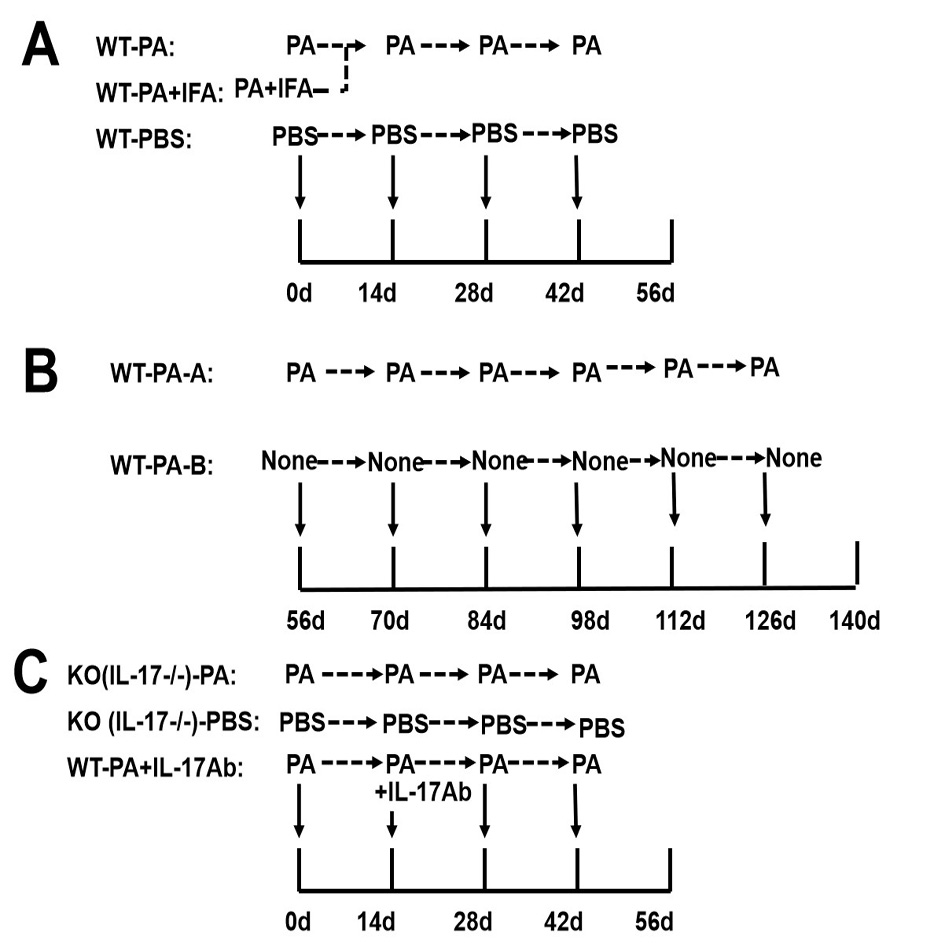
**

**Figure S1 Diagram of the procedure of mouse model development**

1. Procedure for the WT-PA, and WT-PA+IFA, and WT-PBS groups. **(B)** Procedure for the WT-PA-A and WT-PA-B groups. **(C)** Procedure for the IL-17^-/-^-PA (KO-PA), IL-17^-/-^-PBS (KO-PBS), and WT-PA+IL-17Ab. PA: *Propionibacterium acnes*. IFA: Incomplete Freund's adjuvant. KO: knockout.

**
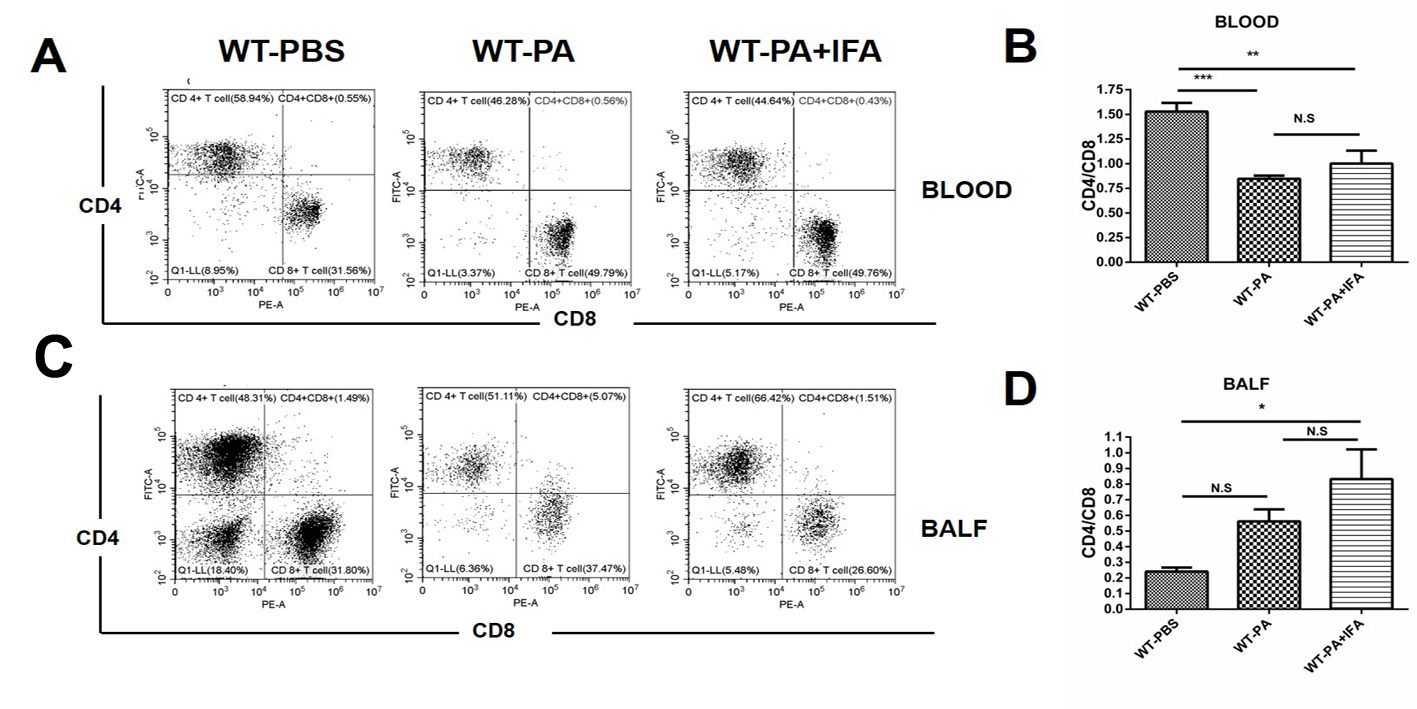
**

**Figure S2 Flow cytometry analysis of mononuclear cells in peripheral blood and BALF samples**

Peripheral blood and BALF samples were collected on the 56^th^ day. Mononuclear cells from peripheral blood sample (**A**) and from BALF **(C)** were analyzed. CD4/CD8 ratio in peripheral blood sample **(B)** and in BALF (**D**) were determined. *P < 0.05.**P < 0.01. ***P < 0.001. ****P < 0.0001. NS, not significant.


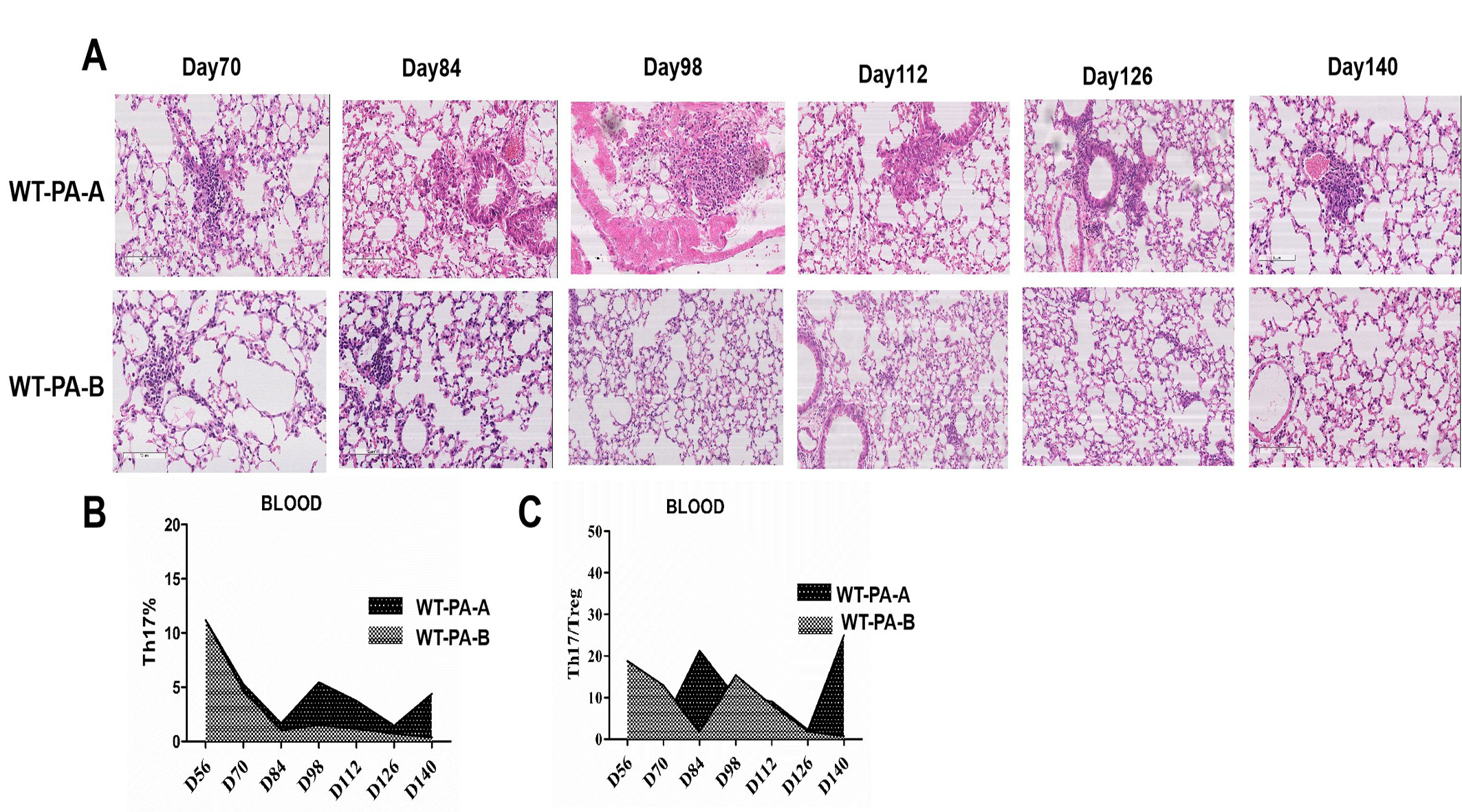


**Figure S3 Histopathological and flow cytometry analysis of samples from the WT-PA-A and WT-PA-B groups**

1. H & E staining. **(B)** Th17%. **(C)** Th17/Treg ratio.

**

FigureS4** **Total cell numbers and Lymphocytes numbers in BALF samples on the 56^th^ day**

**C**

**B**

**D**

**A**

**(A**) Total cell numbers in BALF samples from the WT-PBS, WT-PA, WT-PA+IFA. **(B)** Lymphocytes numbers in BALF samples from the WT-PBS, WT-PA, WT-PA+IFA. **(C)** Total cell numbers in BALF samples from the WT-PBS, WT-PA, KO-PA, KO-PBS and WT-PA+IL-17Ab groups. **(D)** Lymphocytes numbers in BALF samples from the WT-PBS, WT-PA, KO-PA, KO-PBS and WT-PA+IL-17Ab groups.*P < 0.05.**P < 0.01. ***P < 0.001. ****P < 0.0001. NS, not significant.


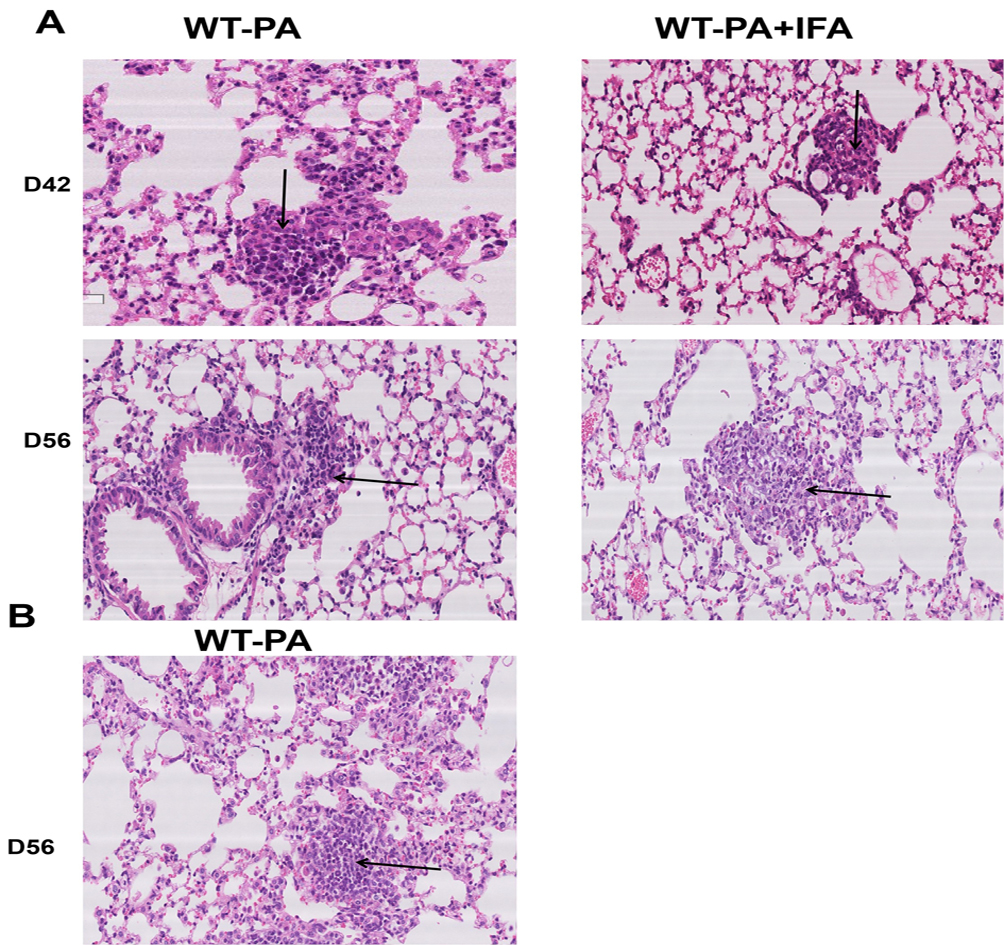


**Figure S5: Higher magnification of the areas with the arrows (in Fig 1 and Fig 4)（300**×**）**

**(A)** Higher magnification of Figure 1 with arrows (300×). **(B)** Higher magnification of Figure 4 with arrows (300×)
